# Effectiveness of an intensive E-mail based intervention in smoking cessation (TABATIC study): study protocol for a randomized controlled trial

**DOI:** 10.1186/1471-2458-13-364

**Published:** 2013-04-18

**Authors:** Laura Díaz-Gete, Elisa Puigdomènech, Elena Mercedes Briones, Mireia Fàbregas-Escurriola, Soraya Fernandez, Jose Luis del Val, Jose Luis Ballvé, Marc Casajuana, Jessica Sánchez-Fondevila, Lourdes Clemente, Carmen Castaño, Carlos Martín-Cantera

**Affiliations:** 1 Centre d’Atenció Primària (CAP) La Sagrera, Institut Català de la Salut, Barcelona, Spain; 2 Primary Healthcare Research Unit of Barcelona, Primary Healthcare University Research Institute IDIAP- Jordi Gol, C/Sardenya 375, Barcelona, entresuelo 08025, Spain; 3 Centre d’Atenció Primària (CAP) La Marina, Institut Català de la Salut, Barcelona, Spain; 4 Centre d’Atenció Primària (CAP) Florida Nord, Institut Català de la Salut, Hospitalet de Llobregat, de Llobregat, Spain; 5 IDIAP- Jordi Gol, Barcelona, Spain; 6 Centro de Salud Santo Grial, Huesca, Spain; 7 La Alamedilla Health Centre, Castilla y León Health Service–SACYL, Salamanca, Spain; 8 Centre d’Atenció Primària (CAP) Passeig de Sant Joan, Institut Català de la Salut, Barcelona, Spain; 9 Departament of Medicine, Universitat Autònoma de Barcelona, Barcelona, Spain

**Keywords:** Smoking cessation, Electronic mail, Clinical trial, Primary health care

## Abstract

**Background:**

Intensive interventions on smoking cessation increase abstinence rates. However, few electronic mail (E-mail) based intensive interventions have been tested in smokers and none in primary care (PC) setting. The aim of the present study is to evaluate the effectiveness of an intensive E-mail based intervention in smokers attending PC services.

**Methods/design:**

Randomized Controlled Multicentric Trial. *Study population*: 1060 smokers aged between 18–70 years from Catalonia, Salamanca and Aragón (Spain) who have and check regularly an E-mail account. Patients will be randomly assigned to control or intervention group. *Intervention*: Six phase intensive intervention with two face to face interviews and four automatically created and personal E-mail patients tracking, if needed other E-mail contacts will be made. Control group will receive a brief advice on smoking cessation. *Outcome measures*: Will be measured at 6 and 12 months after intervention: self reported continuous abstinence (confirmed by cooximetry), point prevalence abstinence, tobacco consumption, evolution of stage according to Prochaska and DiClemente's Stages of Change Model, length of visit, costs for the patient to access Primary Care Center. *Statistical analysis*: Descriptive and logistic and Poisson regression analysis under the intention to treat basis using SPSS v.17.

**Discussion:**

The proposed intervention is an E-mail based intensive intervention in smokers attending primary care. Positive results could be useful to demonstrate a higher percentage of short and long-term abstinence among smokers attended in PC in Spain who regularly use E-mail. Furthermore, this intervention could be helpful in all health services to help smokers to quit.

**Trial Registration:**

Clinical Trials.gov Identifier: NCT01494246.

## Background

Health effects among smokers and non-smokers of tobacco consumption are well-established including principally respiratory and cardiovascular diseases and cancer [1]; in fact is one of the leading preventable causes of death worldwide and mainly in occidental societies [2]. In Spain, for instance, smoking is the health problem that causes more mortality and morbidity [3], and therefore originates one of the higher health costs. According to the last national survey conducted in Spain in 2006 the percentage of daily smokers aged 16 or older was 26.44% [4]. The vast majority of smokers declare a willingness to quit smoking and approximately 60% have tried it [3], although only 3 to 5% of them per year accomplished smoking cessation [3,5,6].

Several cost-effectiveness studies worldwide have shown that both low and high impact care interventions to reduce tobacco consumption are cost-effective measured in cost per year of life gained and quality of life [3,7]. Moreover, under a public health view probably these interventions represent the most cost-effective method to improve population’s health. Minimum advice on quitting smoking among the general population achieves an average of 5% cessation per year [8,9]. In contrast, more intense interventions, can reach more than 20% of abstinence [10,11]; in general, it is well established that the more intensive the intervention, the best rates of smoking cessation are obtained [10]. The inclusion of those treatments that have demonstrated its efficacy in systematic reviews and metanalysis, has been confirmed to be useful [7].

Therapeutic intensive interventions for smoking cessation have been used in primary care, such as the one of the ISTAPS study [12], but none have been based in Information and Communication Technologies (ICTs). These interventions are based on the hypothesis that smokers that do not quit smoking but are aware of its harmful effects underestimate the health risk to due to its consumption [13]. So, these interventions could provide motivational feedback to promote risk awareness and accelerate changes on smoking behavior [14]. Some smokers who quit smoking have been identified as susceptible to damaging adverse effects of tobacco consumption or seen seriously threaten his or her health [15,16].

The vast majority of non-pharmacological use the Prochaska and DiClemente’s Transtheoretical Model of Change [17] that describes a succession of stages (precontemplation, contemplation, preparation, action and maintenance) each one indicating a different type of intervention. Other studies in our country have used this model among diabetic smokers patients attended in primary care and to confirm smoking abstinence using cooximetry [18] and another where information gathered by cooximetry is the main element to motivate smokers to quit [19].

The use of electronic mail and internet is increasing worldwide. In Spain, according to the National Statistics Institute, in 2010, 57% of households had Internet access; moreover there are 20 million estimated regular users and those who log at least once a week represent 58.4% of the population (7.1% increase over the previous year). Seventy-eight percent of the Spanish population between 16 and 74 uses the electronic mail, with an increase of those who access it via mobile devices [20].

Internet and E-mail use is also increasing in medicine and can be mostly useful in appointment citations and remainders, analytical results, computerized medical history, prescription refills, regular follow-up and access to health information. The advantages derived from professional-patient E-mail communication include: time and cost saving for hospitals, primary care centers, primary care professionals and patients (e.g. patients who frequently travel and sanitary professional of rural areas can easily contact with physicians and patients respectively) [21,22] and convenience since it does not have a simultaneous nature (the person can check the messages at his or her convenience) [21]. Several patients also refer that E-mail communication can facilitate the contact with the sanitary professional; in face to face interviews they could not feel enough comfortable to rise certain issues or may have forgotten to ask important questions. Some of them deem a useful and satisfactory use E-mail communication in a secure and private environment [21]. A revision undertaken on the impact of e-therapies, Hsiung concluded that E-mail tracking is useful if preceded by a personal and direct relationship with the patient [23]. Finally, in the global crisis context these types of interventions can diminish visits to the primary care center. Limitations comprise unwillingness to use the E-mail, perception of lack of time and experience to use the new technologies [21,24], an abuse by an overriding number of messages on non urgent matters, and the development of a private and secure website and a specific deontological code could increase the economic costs [25].

Internet use in quitting smoking is becoming more evident, since it is necessary to recruit more smokers to fight smoking regardless of race or culture [26]. Several studies have published about the virtual communities in this field [27] and internet as a support to phone lines (quitlines) [28]. It has been established that it is a way that enriches the sanitary professional-patient relationship and that the dissemination of patient’s successful experiences can improve their self-esteem and generate positive attitudes [25] although further research is needed [29].

The 2008 the Treating Tobacco Use and Dependence Guideline, pointed out that online tools to quit smoking are promising tools that have not achieved its own potential to undertake interventions; results have been shown to be only favorable if online interventions are developed in very complex programs or if were carried out when the help was more intensive among intervention groups than in controls. Nevertheless, it also encourages depending on those characteristics that needs to be improved [7]. In Spain, the National Committee to Prevent Smoking Cessation (CNPT in Spanish) also supports these types of interventions to help smokers to quit [3].

Characteristics of smokers who seek help on the Internet to quit smoking were female sex (59%) and younger individuals. Generally are subjects who are trying to quit smoking (53%) and search information on how or medication to quit, whilst those who have already quit search information on how to face abstinence [30].

Twenty studies have been found to be registered at Clinical Trial when using keywords: E-mail AND smoking, none undertaken in Primary Care environment with personalized follow-up (Clinical Trials, 2012). In 2010 the Cochrane library published a revision that included the most relevant articles on internet interventions for quitting smoking. Ten out of twenty compared an internet based intervention with others not based on this media. The revision concluded that personalized and interactive internet based interventions, especially if there is an individualized tracking, can be more effective than those standardized protocols on smoking cessation. However, long-term smoking cessation is less effective when using these interventions compared to other interventions [31].

Te Poel and collaborators published in 2009 a clinical essay to evaluate the efficacy of an E-mail computer-tailored smoking cessation intervention. At six months post intervention 21.5% and 20.4% of intervention group reported not having smoked in the last 24 hours and in the 7 days, respectively compared to the control group (9.8 and 7.8%, respectively); follow-up lost were quite high in both control and intervention groups [32]. Polosa et al. demonstrated the utility of an E-mail consultation messages in a smoking-cessation program although the number of included patients was somewhat low [33]. Lenert and collaborators determined the effectiveness of an automated educational E-mail messaging system individually sent in the framework of smoking cessation intervention. The OR for quitting smoking among the intervention group at 30 days post-intervention was 2.6 (95% CI: 1.3-5.3) [34]. An increased smoking and reduced frequency of smoking was observed in young adults after an online peer support via E-mail [35] and in adolescents after a home based internet intervention [36,37].

In Spain there are two online initiatives to help smokers to quit. The first one, the Madrid City Council Giving-up smoking on-line Programme, that combined automated interaction with the user and personal E-mail consultations that included 4865 smokers. After the treatment period the smoking cessation rate was 17.8% and at six months post intervention the abstinence achieved was 10% [38]. The second one is undertaken by the Psychology Faculty of the National Distance Education University (UNED) but not results have been published yet [39].

In Barcelona, Spain, the study *E*-*consulta* in Primary Care setting is being undertaken. Its main objective is to develop a secure web environment for sanitary professional-patient communication via E-mail (e.g. acute health problems, laboratory results, health measurement reports, or prescription refills) to avoid unnecessary face to face visits. The sanitary professional committed to answer patients E-mail on the following 48 working hours. The study is carried out in twelve primary care centers and includes 19 physicians, 16 nurses and 647 patients. This initiative has been positively evaluated by both sanitary professionals and patients and it is planned to spread it to all territorial setting [40].

Both, The *E*-*consulta* and the experience described by Wallwiener and collaborators in 2009 [22,40], allow to think that better service and satisfaction of primary care services will be achieved by reducing resources of primary care addressed to smoking cessation. If sanitary systems facilitated E-mail based interventions on smoking cessation, and other sanitary problems, costs could be reduced and medical care and patient and sanitary professional satisfaction could be improved.

## Methods/Design

### Study design

A randomized controlled multicentric trial to evaluate the effectiveness and the cost effectiveness of a clinical practice guide based intervention, based in six contacts (two face to face interviews and 4 E-mails), to obtain continued smoking abstinence at 6 and 12 months compared to brief advice in smokers attended in Primary Care.

### Study population

Inclusion criteria of participants will comprise: smokers (of at least one cigarette per day) aged between 18 and 70 years who have an electronic mail account and check it regularly (at least once a week) and can assure their participation in the study for a year.

Exclusion criteria will be based on certain medical conditions which could contraindicate the fulfillment of the intervention such as known terminal illnesses, severe mental diseases, addiction to other psychoactive substances, patients that have already started to quit smoking or those unwilling to participate in the study were excluded. Smokers who do not have an E-mail account or do no not check it regularly will also be excluded, as well as those smokers who do not wish to be followed via E-mail as prefer face to face contacts.

Written informed consent will be obtained from all subjects of both intervention (IG) and control group (CG) prior to its inclusion on the study.

### Recruiting process

#### Inclusion of sanitary professionals

Diffusion of the project will be made in all primary care centers of the study area through the Xarxa de Centres Sense Fum (network of smoke free centers) to obtain sanitary professionals (general practitioners and nurses) interested in recruiting patients for the study. Of those willing to participate we will select only those who are regular users of E-mail and will be asked to sign a participation commitment. Causes of refusal to participate will be recorded. Those professionals interested in participating will attend a two-hour formative activity where the aim, methods, intervention will be explained and will be trained in the management of project’s web page and cooximetry technique. Assistance to one of the ten formative activities scheduled at different times to facilitate professional attending will be mandatory to participate in the study.

#### Inclusion of participants and randomization

**Figure 1:**
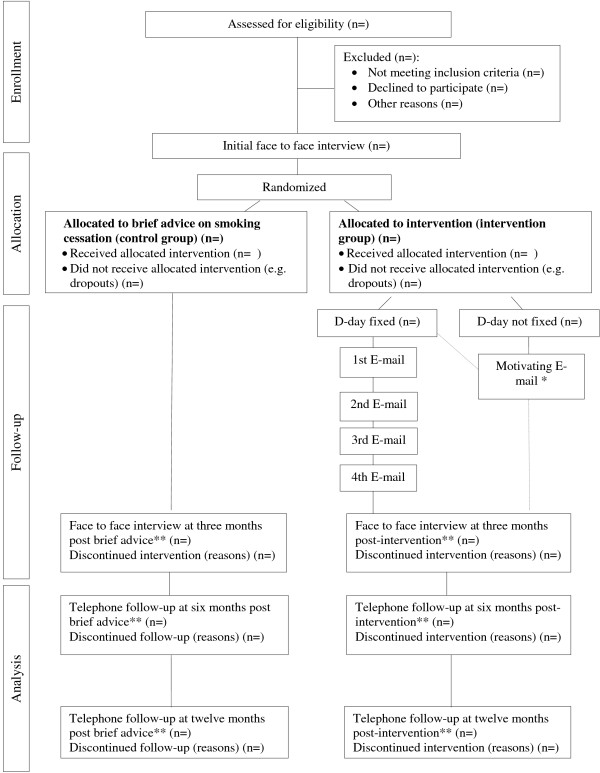
**Algorithm of the TABATIC study.** ** A confirmatory cooximetry in standard conditions will be done on those subjects that declare tobacco abstinence. * Those individuals who are not willing to fix the D-day (usually in contemplative and precontemplative stages) will be contacted only once via E-mail after a month of the first face to face interview to encourage them to quit smoking. The patient will be able to fix the D-day up to 15 days prior to the second face to face interview.

During the recruitment period (first semester of 2013), trained professionals of each primary health care centre (general practitioners or nurses) will invite to participate subjects who meet the inclusion criteria by random systematic procedures. Twice a week each professional will invite to participate the first two smokers aged 18 to 70 who consulted them for any reason. In order to follow the criteria specified in the CONSORT Guide, we will register for all the subjects invited to participate (included or not in the study) on the web page of the study: date of interview, professional who realizes the visit, age and sex of the participant, and consumption of tobacco. Causes of refusal to participate will be recorded. Written informed consent to participate in the present study will be asked to all the individuals who accept to participate. Afterwards, the system of randomization of patients in control and intervention group (1:1 ratio) will be centralized and computer generated. Figure 1 shows the flow chart of the study.

### Description of the intervention

#### Control group

Subjects assigned to the control group, once the motivating problem of the clinical visit had been solved, will receive a brief advice on quitting smoking. Three months after the intervention a second face to face interview will be done to this group to check tobacco abstinence.

#### Intervention group

The proposed intervention will consist in the application of the recommendations of Miltenberger [41] and an evidence-based practical clinical guideline of the Primary Care Division of the Catalan Institute of Health [42]. Both propose an intervention previous to the day the patient sets to stop smoking (D-day) to reinforce behavior, to follow the patient a week after the D-day since approximately 25% of smokers who attempt to quit relapse within the first week and an E-mail feedback to reinforce behavior, to praise positive achievements (such as cigarette consumption reduction) and provide simple corrective feedback when the patient does not perform the target behavior. The basic steps on the intervention are:

1. First face to face interview: In the first contact with the patient, sociodemographic and related to tobacco consumption variables will be collected using a structured face to face interview. E-mail tracking will be approximately set for those individuals in the preparation/action stage, according to the model defined by Prochaska and DiClemente.

2. First E-mail contact: Will be done the following day from the first face to face interview in order to establish the day the individual will stop smoking (D-day), the treatment (if needed), to assess and facilitate motivational support to achieve individual’s goal to quit smoking.

3. Second E-mail tracking: Will be done a week later of D-day to evaluate abstinence, tolerance and adverse effects of medication, difficulties in everyday life, and to control medication.

4. Third E-mail tracking: Will be done 10 to 15 days after D-day to evaluate abstinence, gained benefits, to prevent relapsed and to control medication.

5. Fourth E-mail tracking: Will be done a month after D-day to prevent relapsed and false securities, to evaluate obtained benefits and to control medication.

6. Second face to face interview: Will be done three months after D-day to finish the treatment, to emphasize the effort made and improvement in patient conditions and to stress and prevent possible relapses. On those who declare tobacco abstinence an analysis of exhaled carbon monoxide (CO) will be made by cooximetry in standard conditions to confirm it.

The six phases of the intervention will be made on those patients who fix the D-day by the same sanitary professional who made the first contact with the patient and usually is the one who gives him/her attention, independently if he or she is a general practitioner or a nurse. E-mail messages will be automatically created by an automatic system and will clearly specify the name of the patient, information on the visits, the D-day (if fixed) and the name of the sanitary professional. Besides, the above established E-mail tracking, other E-mail contacts will be made, if needed, in order to clarify subject’s possible doubts (such as medication queries). Nature of the E-mails of the intervention o the study is offered in Additional file 1.

Those individuals who are not willing to fix the D-day (usually in contemplative and precontemplative stages) will be contacted only once via E-mail after a month of the first face to face interview to encourage them to quit smoking. A specific E-mail message will be sent to those who refer a lapse. The patient will be able to fix the D-day up to 15 days prior to the second face to face interview.

The study web page will be developed and maintained by an external company that will also offer support during the intervention process. In order to ensure confidentiality, we will assign to each participant his/her user identification and password so he or she will be able to read the electronic messages and reply if necessary. Sanitary professionals will also have their own identification code and password and will have only access to their own patient’s information. Moreover the study web page will be accredited as secure web environment in accordance to the national current legislation.

Both groups, control and intervention, will be followed at six and twelve months after the intervention by phone. The calls will be made by trained monitors of an external company who will not know if the patient belongs to control or intervention group, and will include a brief structured questionnaire to know their tobacco consumption, their stage of change and tobacco abstinence. A confirmatory cooximetry in standard conditions will be done on those subjects that declare tobacco abstinence.

### Outcome measures

#### Main outcome measure

Self reported ‘continuous abstinence’ defined by Hughes et al. 2003 [43] as *the abstinence between quit day and a follow*-*up time* (at six and twelve months) after intervention confirmed by a breath CO cooximetry concentration of 6 parts per million (ppm) or less in standard conditions.

*Other outcome measures*:

– Point prevalence abstinence according to Hughes et al. 2003 [43] defined as *prevalence of abstinence during a time window immediately preceeding follow*-*up* at three, six and twelve months after intervention confirmed by breath carbon monoxide cooximetry in standard conditions.

Self reported tobacco consumption at three, six and twelve months after intervention: daily consumption in cigarettes/day.

– Self-reported reduction of daily tobacco consumption in cigarettes/day when the subject does not stop smoking at three, six and twelve months after intervention.

– Change of stage in the Prochaska and DiClemente's Stages of Change Model at three, six and twelve months after intervention.

– Prescription of tranquilizers, antidepressants, NRT (nicotine replacement therapy), at the beginning and end of the intervention and six and twelve months post intervention.

– Registered use of sanitary services in primary and specialized care, both in public and private settings.

– Time used by professionals in the two face to face interviews (initial and three months visits).

– Time used by sanitary professionals in case they have to answer E-mail contacts.

– Time used by patients in case they have to ask questions during the E-mail intervention.

– Costs for the patients to access Primary Care service at three, six and twelve months after intervention.

*Independents*:

– Type of intervention: Intensive counseling by E-mail vs brief advice.

### Data collection

At baseline visit, face to face interview with the sanitary professional will be carried out and the following information will be registered:

– Socio-demographic characteristics: age, gender, civil status, educational level, social class according to the classification of the UK Registrar General's social classification (RGSC).

– E-mail frequently used and data for the telephone tracking.

Characteristics of individual's tobacco consumption: daily consumption in cigarettes/day, years of smoking, age at the start, time before first cigarette of the day, nicotine, dependence measured by the Fagerström test [44], number of previous attempts to quit smoking, maximum time of abstinence, pharmacological treatment used on previous attempts to quit smoking: type, timetable, side effects, family and friends environmental tobacco consumption, partner interaction questionnaire (PIQ Test) to measure of spouse/partner support related to cessation [45].

– Change of stage in the Prochaska and DiClemente's Stages of Change Model.

– Alcohol consumption.

– Morbidity (related or not to tobacco consumption): chronic obstructive pulmonary disease, cerebrovascular event, ischemic cardiopathy, arterial hypertension, hypercholesterolemia, cancer and peripherical arteriopathy, diabetes mellitus, obesity.

– Weight.

– Breath carbon monoxide (CO) in ppm measured by cooximetry which will be carried out by trained health professionals using following a pre-established protocol.

At three months after intervention a second face to face interview will be undertaken with the sanitary professional and the following information will be recorded: time spent in the visit and if the subject currently smokes. If so, daily consumption in cigarettes/day and stage of change according to Prochaska and DiClemente's Stages of Change Model will be recorded. If not, date of smoking cessation, time (in days) of abstinence will be asked, and level of breath CO by cooximetry in standard conditions. Self-reported weight will be documented in both cases. Date of visit and the professional who undertakes it will be additionally recorded.

At six and twelve months after intervention all participants will be asked by phone about some variables related to tobacco consumption and stage of change in the Prochaska and DiClemente's Stages of Change Model (described above) by trained monitors. Moreover, at twelve months after intervention, level of satisfaction, acceptance of intervention, number of E-mail messages written and number of replies and level of social support during the intervention measured by PIQ Test will be recorded.

### Data management and quality assurance

In order to guarantee and to ensure the quality of the study, data to maximize validity and reliability the following measures will be employed:

•Written documentation and electronic data collection: Written copies of protocols and consent forms will be printed and stored in the study web page. All data will be registered in the electronic data collection system developed and maintained specifically for this study by an external company to assure consistency. Regular backups will be performed and transferred to the central database as well as random checks of data entry. If needed, corrections will be made by checking paper records or, in rare cases, by phoning participants for confirmation by independent investigators.

•In order to guarantee the correct registration of data, a training session will be made to all sanitary professionals who participate in the study in the intervention and the use of the electronic data collection system. The contents of the training sessions will be evaluated by completing a standardized evaluation form to ensure the consistency of the program.

•Regular meetings and mailings between members of the study group (the TABATIC team) and all participating centers. Furthermore, a person has been contracted part-time to provide technical and methodological support to all investigators of the study via E-mail or telephone.

•Telephone interviewers will be trained on characteristics and procedures of the study.

### Sample size

Accepting an alpha risk of 0.05 and a beta risk of 0.20 in a bilateral contrast, 1060 individuals are needed; 530 individuals in the IG and 530 in the CG in order to detect a difference of at least a 5% among both groups. A continued abstinence of 5% was assumed for the non intervention group and a dropout rate of 20% is estimated. The sample size calculations were performed with the Granmo program (version 7.1).

### Statistical methods

Data will be analyzed in concordance with the Consort Cluster guide [46], and all analyses will be done on an intention to treat basis. Baseline descriptive statistics among CG and IG in relation to the variables studied will be computed as customary; in contingency tables Pearson’s Chi-square test for independence or homogeneity will be applied to assess the relationship between two categorical variables, T-Student test or ANOVA will be used in the comparison of means if the variables follow a normal distribution and U of Mann Whitney test if they do not. For the other dimensions of the analysis, a covariance analysis (ANCOVA) for repeated measures will be carried out.

A multilevel logistic regression and a Poisson regression will be done to evaluate the association between each of the dependent variables and the independent variables that have resulted statistically significant in the bivariate analysis. All analysis will be adjusted for potential confounding factors and for variables with clinical relevance. The level of statistical significance will be set at 0.05, and all tests will be two-tailed. Statistical analyses will be conducted using SPSS, version 17.0 (SPSS Inc, Chicago, IL).

### Cost analysis

A cost-effectiveness analysis will be also undertaken under a societal perspective to compare the cost of the new intervention versus the usual care, to know the clinical effectiveness and the resource savings for the National Health System [47]. Subsequently, a deterministic sensitivity analysis will be performed to assess the robustness of the results [48].

### Ethical approval

This study has been reviewed and approved by the Clinical Investigation Ethics Committee of the IDIAP Jordi Gol, located in Barcelona, Spain and registered at Clinical Trials (code number: P11/41). The participation in this study is strictly voluntary and withdrawal will not have any consequence on the management of the subject illness, which will be carried out rigorously following the accepted international norms. The data will be treated confidentiality according to the Organic Law which regulates the confidentiality of computerized data (Personal data Protection Law 15/1999); only the investigators and monitors/auditors of the study will have access to the data of the subjects who agreed to participate.

### Associated studies

We are currently undertaking two associated studies to the TABATIC Project. The first, a descriptive one aims to know the availability and use of Communication and Information Technologies among smokers attended in primary care. The second one, a qualitative, intends to find out barriers and facilitators of Communication and Information Technologies to help smokers to quit, that both, smokers and health professionals who help patients to quit smoking, refer.

## Discussion

The proposed intense intervention consists in the application of the recommendations of an evidence-based practical clinical guideline of the Primary Care Division of the Catalan Institute of Health [42] based in six contacts (two face to face interviews and 4 E-mails), to obtain continued smoking abstinence at 6 and 12 months. There are some international studies that use E-mail based interventions to help smokers to quit, but none has the same characteristics as the one proposed [5,10,26,27,32-35,37,49]. Furthermore, to our best knowledge, in Spain there are no studies that use this type of interventions at any level of clinical services.

Since the implementation in 2006 of the law 28/2006 for smoking prevention, the number of primary care consultations regarding smoking cessation has been raised, subsequently an approach to smoking cessation should be a key essential objective of both the clinical practice and public health research. Subsequently, primary care is an ideal setting to develop a health counseling E-mail program based on a practical clinical guideline due to its almost universal coverage in Spain [3,50]; it is estimated that 75% of the population visits his or her general practitioner at least once a year, and that people who smoke do so more often than non-smokers [3,51].

One of the main strengths of the TABATIC project is that, to our best knowledge, for the first time the therapeutic use of E-mail tracking will be evaluated in order to help smokers to quit. Furthermore, the same sanitary professional, who habitually attends the patient and with whom a previous confidence relationship has been established, is the one who follows the patient during this intervention which can lead to more successful outcomes if compared to other more impersonal follow-up interventions such as web pages or community programs [31]. Another strength of E-mail tracking interventions according to Kuppersmith is its *asynchronous nature*; E-mail communication allows physician and patient not to be available at the same time. In addition, both can access at their messages at their own convenience and as many times as needed [21]. Moreover, this type of intervention could reduce social and sanitary costs due to its great accessibility, its capability to diminish trips and timeouts to be attended.

Selection bias among health care professionals may occur since those who volunteered to participate could be more motivated than other sanitary professionals. Randomization will be made among smokers and not in primary care centers which will lead to use this E-mail intervention in only some of his or her patients; however the randomization will be previously automatically created so the professional will not be able to decide on which patients should use the intervention since it will be computer generated. Patients assigned to the control group will receive a brief advice on quitting smoking. One of the main limitations of the study is that we will only include professionals who know how to use computers and E-mail and can assure participation in the study by using it. However we feel confident that an important number of participating professionals will run correctly the program since most of them have previously participated in other complex tobacco studies undertaken in our territory such as the ISTAPS (176 basic care units that worked in 82 Spanish primary care centers; participants: 2,827 smokers aged 14–85 years), ITADI (400 professionals and 1077 diabetic smokers) and BIBE studies (162 pediatric teams from 96 Spanish primary care centers; participants: 1101 child) [12,18,52]. No economic compensation will be offered to sanitary professionals; consequently, those who expect to be reimbursed for this extra activity will be also excluded of the study. Some professionals may be reluctant to use electronic mail in professional health care-patient communication. Reasons to be reluctant may include the believe that written messages can omit important connotations in communication (such as body language or voice tone), the concern that individuals may send a vast number of irrelevant messages and the possible dissemination of health professional e-mail address [21,53]. To assure health care professional and patient confidentiality, an external company will develop and maintain the study a secure web page; if needed, they will control and limit each subject web page use [54]. We are currently carrying out a qualitative study in order to describe possible barriers and facilitators of Information and Communication and Technologies to help smokers to quit.

If the proposed intervention is effective, it should be adapted to the informatics systems of each local health service.

The present trial aims to show the effectiveness of an E-mail smoking cessation intervention based in the recommendations of an evidence-based clinical practice guideline of the Primary Care Division of the Catalan Institute of Health at six and twelve months post-intervention [42]. The goal is to propose a new and effective intervention in primary care, as well as in other sanitary settings, as a complementary tool to treat smokers.

## Abbreviations

CG: Control group; E-mail: electronic mail; ICT: Information and Communication Technologies; IG: Intervention group.

## Competing interests

The study authors declare that they have no competing interests.

## Authors’ contributions

LDG and CMC were responsible for the conception and design of the study and wrote the firsts drafts of the study protocol. LDG, CMC and EPP conceived and participated in the design of the questionnaires and wrote the final version of the study protocol. All other authors contributed to the design and development of the study and the questionnaires, as well as to the writing and development of the protocol. EPP, LDG and CMC wrote the first drafts and the final version of this manuscript. All authors have performed a critical revision of this manuscript and the final version.

## Pre-publication history

The pre-publication history for this paper can be accessed here:

http://www.biomedcentral.com/1471-2458/13/364/prepub

## Supplementary Material

Additional file 1 Nature of the E-mails of the intervention of the TABATIC study. Click here for file
