# Supplementary material for: Effectiveness of an intensive E-mail based intervention in smoking cessation (TABATIC study): study protocol for a randomized controlled trial
Source: BMC Public Health. 2013 Apr 18;13:364. doi: 10.1186/1471-2458-13-364 (PMC3648415; doi:10.1186/1471-2458-13-364)
Supplement: Additional file 1 — Nature of the E-mails of the intervention of the TABATIC study. [file 1471-2458-13-364-S1.doc]

Additional file 1. Nature of the E-mails of the intervention of the TABATIC study

Dear sir/madam xxxxxxxx *

According to the visit done on xx/xx/xx *(date of first visit) you fixed your quitting date on: xx/xx/xx *. Seven days have passed, so I would like you to answer the following questions regarding tobacco consumption:

Have you successfully quit smoking? (Yes/No)

If yes, have you experienced difficulties to quit smoking? (Yes/No)

Please, rate from 1 to 5 the difficulties (being 1 no difficult and 5 very difficult) you have experienced on the quitting smoking process: (numeric field)

If you are currently smoking, how many cigarettes per day do you smoke? (open numeric space)

If you are taking medicine to stop smoking, do you have any problems with it? (Yes/No)

Please do not hesitate to ask any questions through this internal messaging (open field).

Shortly you will receive my notes/comments.

Thank you.

Xxxxxxxx * (name of the doctor or nurse)

Fields * are automatically generated by the web system, based on the data provided by patients and professionals when included in the study.
